# 伴MAP2K1基因突变的经典型毛细胞白血病1例

**DOI:** 10.3760/cma.j.issn.0253-2727.2022.05.016

**Published:** 2022-05

**Authors:** 冲 魏, 昊 蔡, 显勇 蒋, 玄 汪, 道斌 周, 俊玲 庄

**Affiliations:** 中国医学科学院、北京协和医学院北京协和医院血液内科，北京 100730 Department of Hematology, Peking Union Medical College Hospital, Chinese Academy of Medical Sciences & Peking Union Medical College, Beijing 100730, China

患者，男，59岁，于2020年9月体检查血常规：WBC 2.63×10^9^/L，中性粒细胞计数（ANC）0.50×10^9^/L，HGB 91 g/L，PLT 63×10^9^/L；腹部超声示脾大。患者未就诊。2020年12月患者出现乏力、活动耐量下降，伴盗汗，体重2个月内下降10 kg。2021年2月7日患者误伤手指后出现反复发热，体温最高39.2 °C。就诊我院急诊，查血常规：WBC 0.55×10^9^/L，ANC 0.11×10^9^/L，HGB 51 g/L，PLT 45×10^9^/L；血EB病毒DNA 2 400 拷贝/ml；支原体抗体1∶160（+）；CT示双下肺间质性改变，纵隔及腹主动脉旁淋巴结肿大，脾大；血培养肠炎沙门菌阳性。诊断为肠炎沙门菌血流感染、支原体肺炎，予美罗培南、莫西沙星抗感染治疗，10 d后体温正常。转入血液科排查全血细胞减少病因。血涂片未见异常；骨髓象：可见毛细胞，占32.5％，该细胞边缘呈“毛发”样突起，或呈“伪足”状、撕扯状；酸性磷酸酶染色阳性率95％，不被酒石酸抑制（TRAP阳性）（图1）。骨髓免疫分型：B细胞占淋巴细胞51％，主要表达CD19、CD20、CD22、CD11c、CD103、CD25，部分表达FMC7、CD23、CD200、kappa、sIgM；不表达CD5、CD10、CD138、CD38、lambda；骨髓活检：造血组织比例明显增多，结合免疫组化，符合B细胞淋巴瘤；免疫组化：CD20（+），CD79b（+），CD5（−），CD10（−），CyclinD1（−），BRAF V600E（−）；二代测序检出MAP2K1基因 I103N、Y130N位点突变，均为致病突变。IgHV区重排检测检出一群寡克隆IgH重排细胞，占B细胞的99.25％，选用IGHV3-11，V区突变阳性。PET-CT：多发代谢增高淋巴结（最大者2.0 cm×2.7 cm，SUVmax 5.5），肝右叶局灶性代谢增高灶（SUVmax 3.6），脾大伴代谢弥漫不均匀增高（SUVmax 4.5），中轴骨、双侧肱骨及股骨骨髓代谢不均匀弥漫性增高（SUVmax 3.8）。依据典型骨髓形态学及免疫表型，该患者诊断为经典型毛细胞白血病（HCL）。于2021年3月11日开始化疗（克拉屈滨10 mg/d，×5 d）。化疗第1天即出现发热，伴左小腿皮肤红肿、疼痛，皮下波动性包块，抽脓液送检细菌培养阴性，先后予头孢美唑、替考拉宁、利奈唑胺抗感染治疗，发热缓解，左小腿肿痛好转。2021年3月27日患者出现发热、气促，伴低氧，血气检查示Ⅰ型呼吸衰竭，胸部CT示双肺弥漫磨玻璃影，送检病原学检查包括痰细菌、真菌培养、六胺银染色、抗酸染色、卡氏肺孢子菌（PCP）-DNA、血CMV-DNA均阴性，结合临床表现及影像学，考虑PCP不除外，因患者存在磺胺过敏史，故加用卡泊芬净抗感染，并联合甲泼尼龙40 mg每日两次，1周后复查胸部CT较前明显好转，1个月内甲泼尼龙减停。患者于化疗后第26天脱离粒细胞缺乏及血细胞输注，化疗后第40天血常规恢复正常，4个月后复查骨髓象示完全缓解，流式细胞术检测微小残留病（MRD）为0％。此时左小腿皮肤破溃伴窦道形成，予换药处置，至2021年9月皮肤破溃完全愈合。随访至今血细胞正常，外周血未见异常细胞，B超检查脾脏正常。

**图1:**
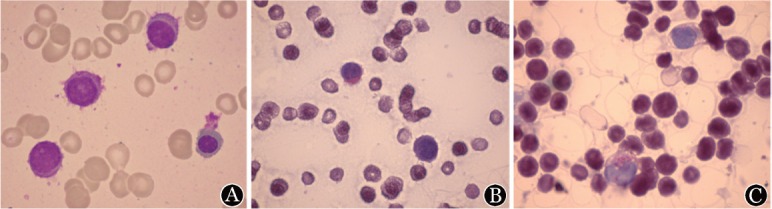
患者骨髓涂片 A：骨髓中可见边缘呈“毛发”样突起的毛细胞（瑞氏-吉姆萨染色，×1000）；B：酸性磷酸酶染色阳性率为95％；C：不被酒石酸抑制

